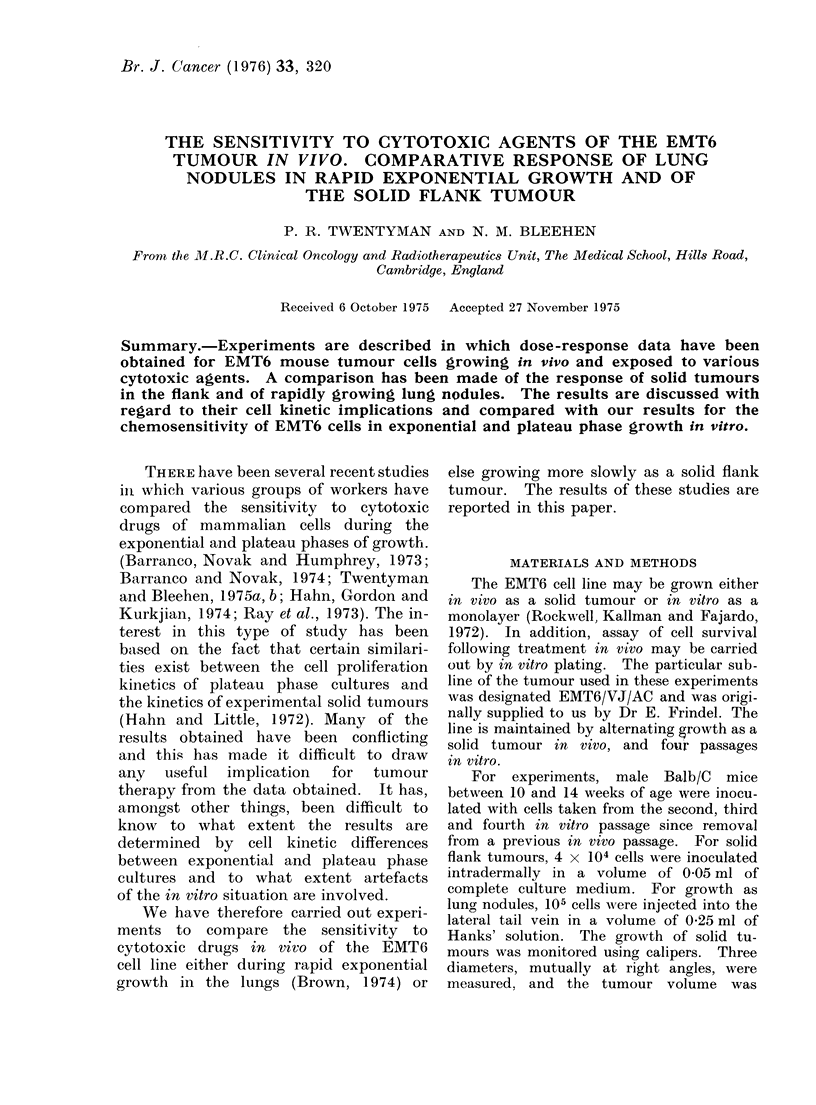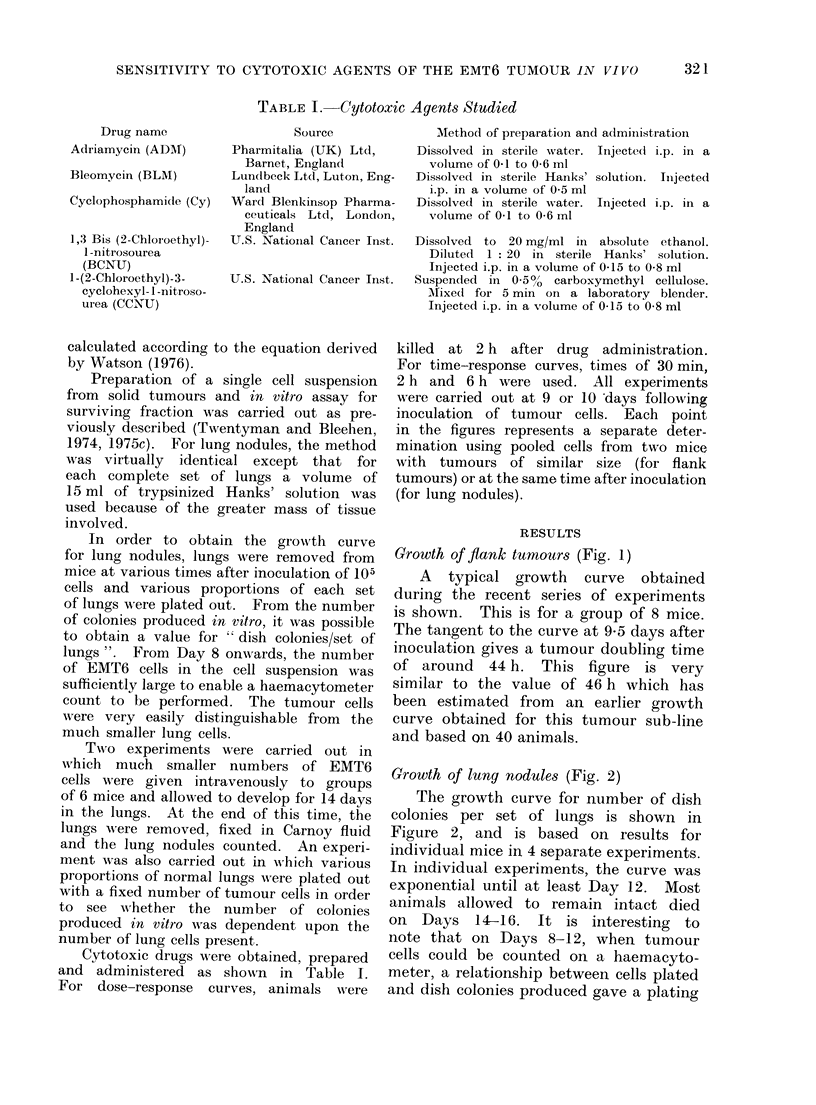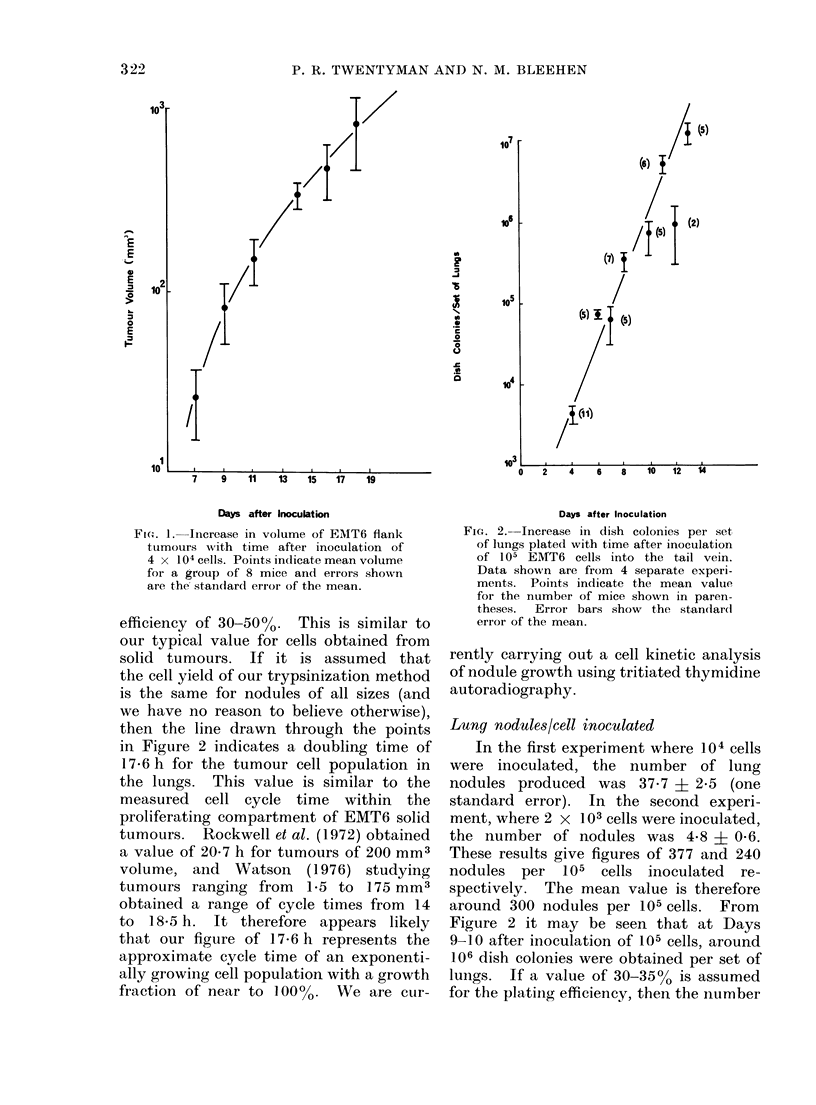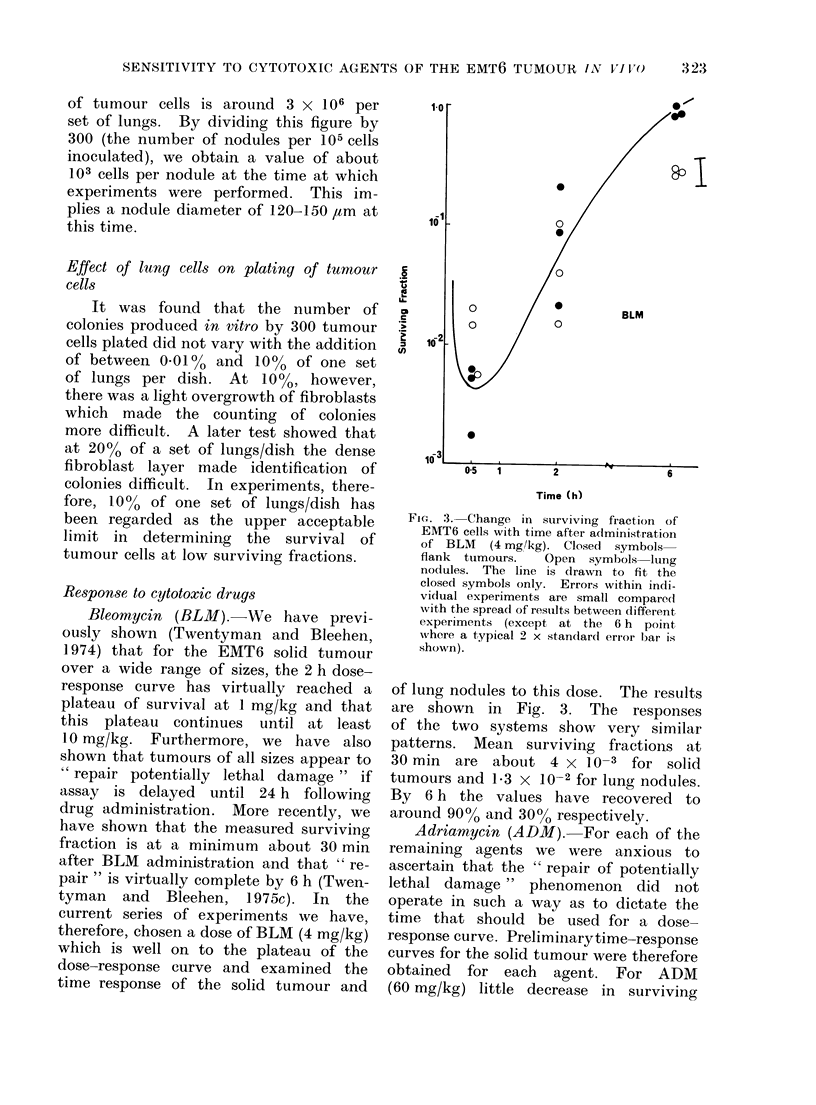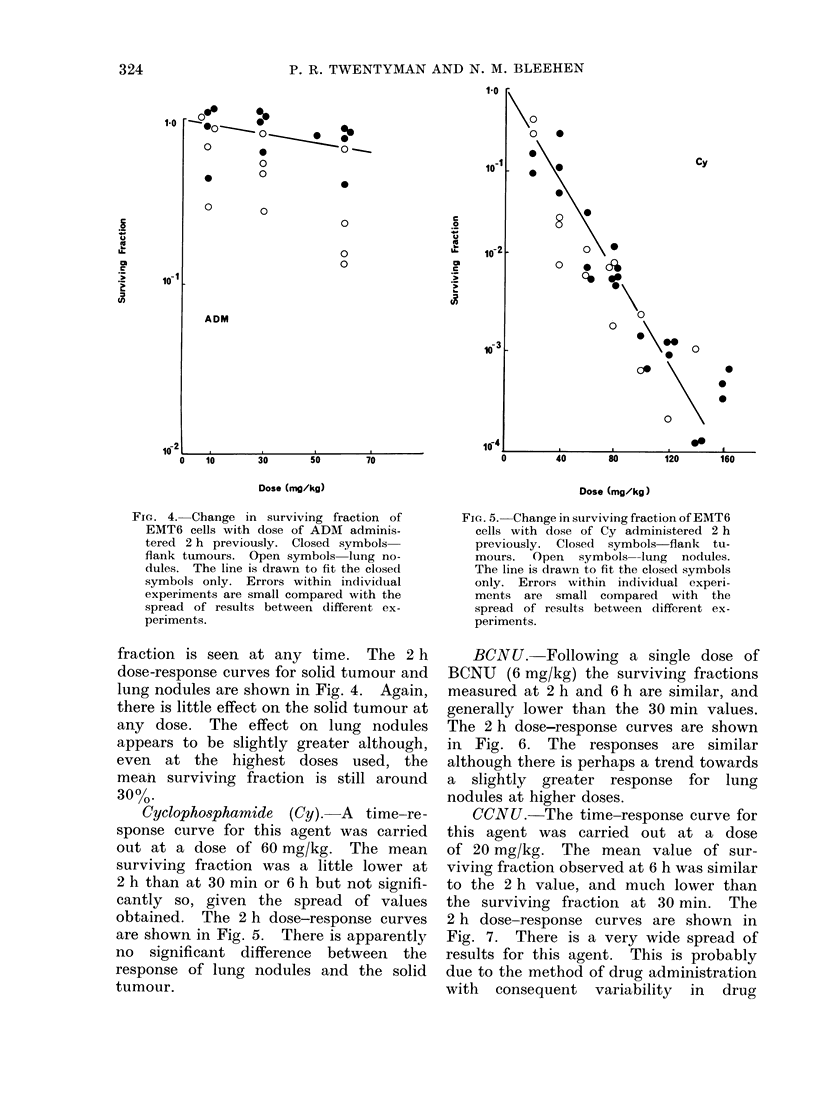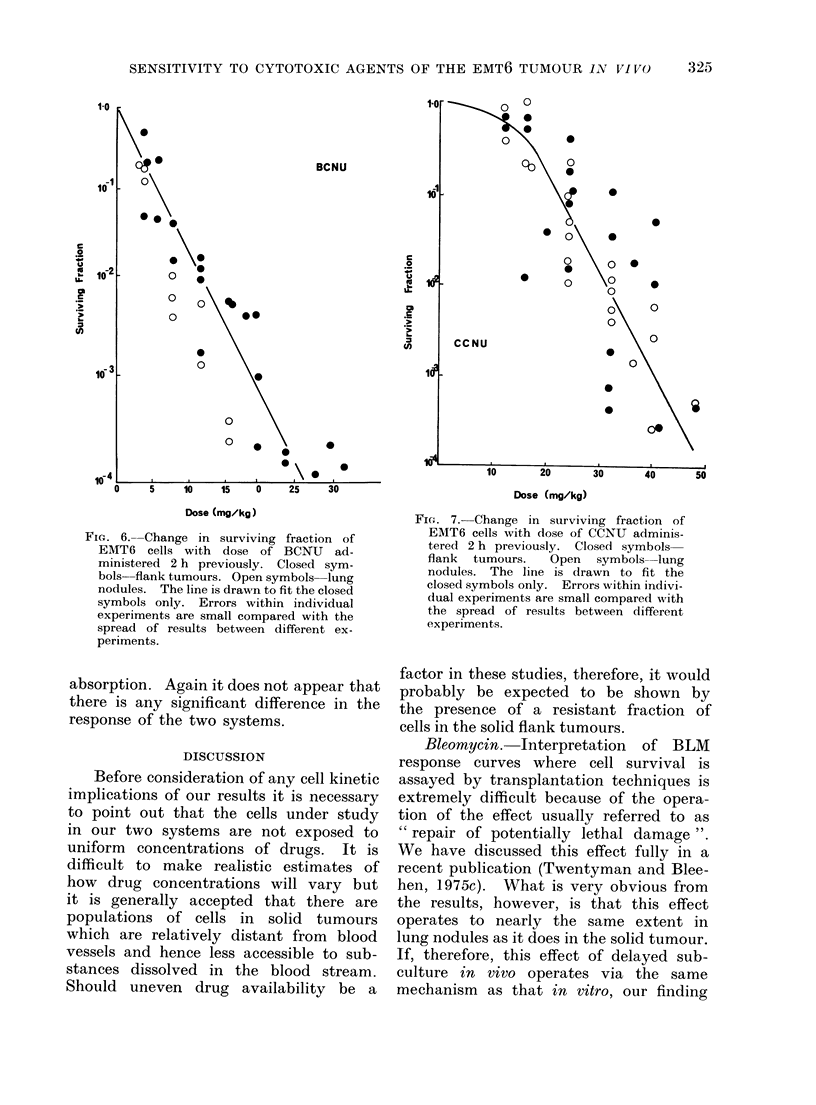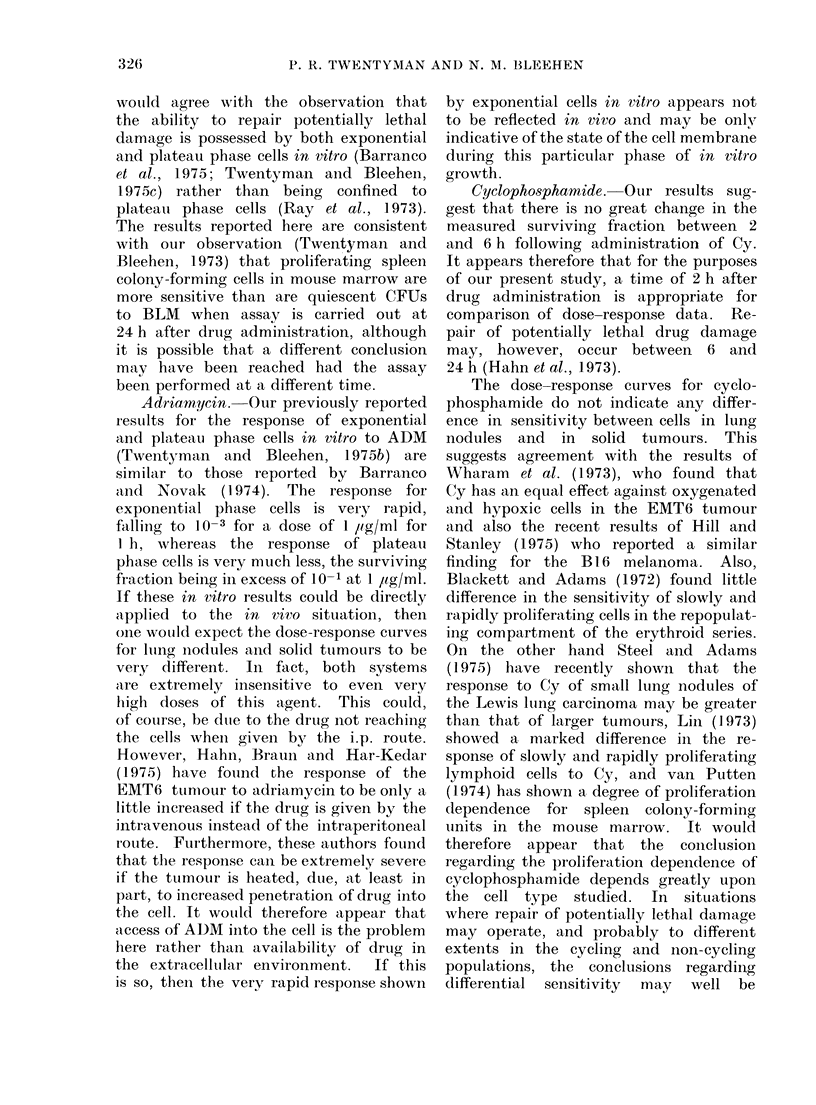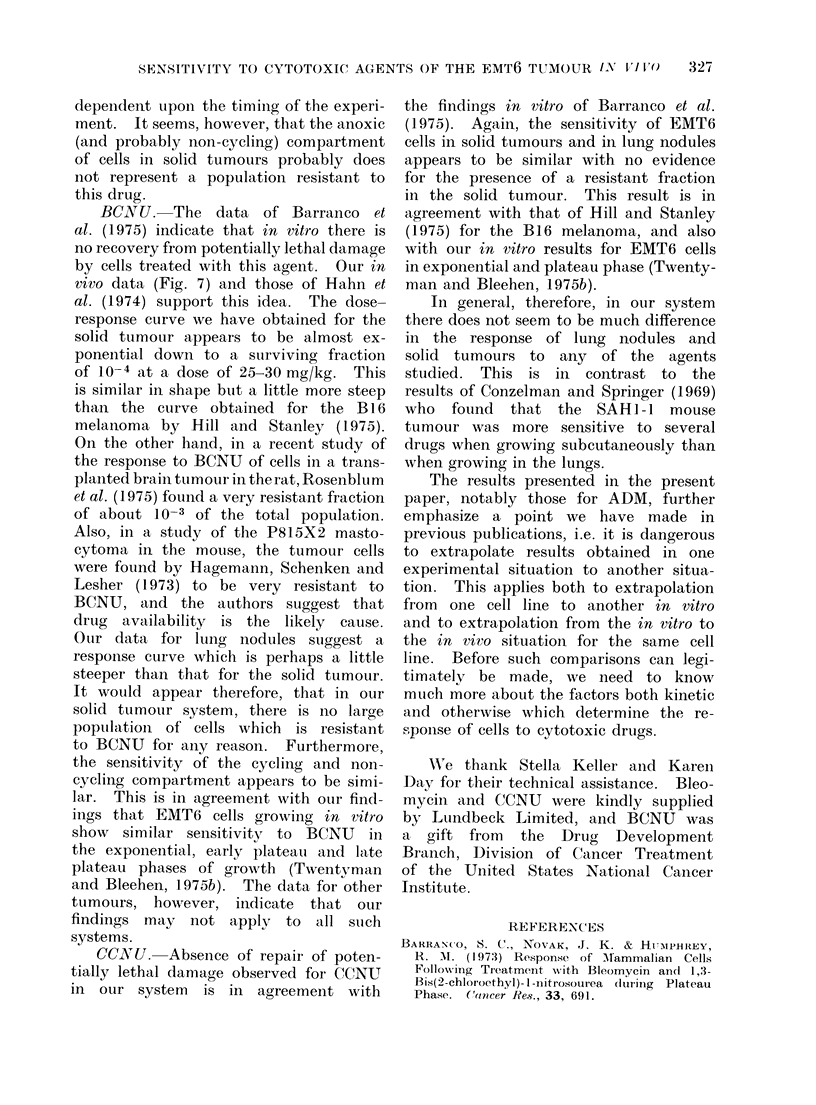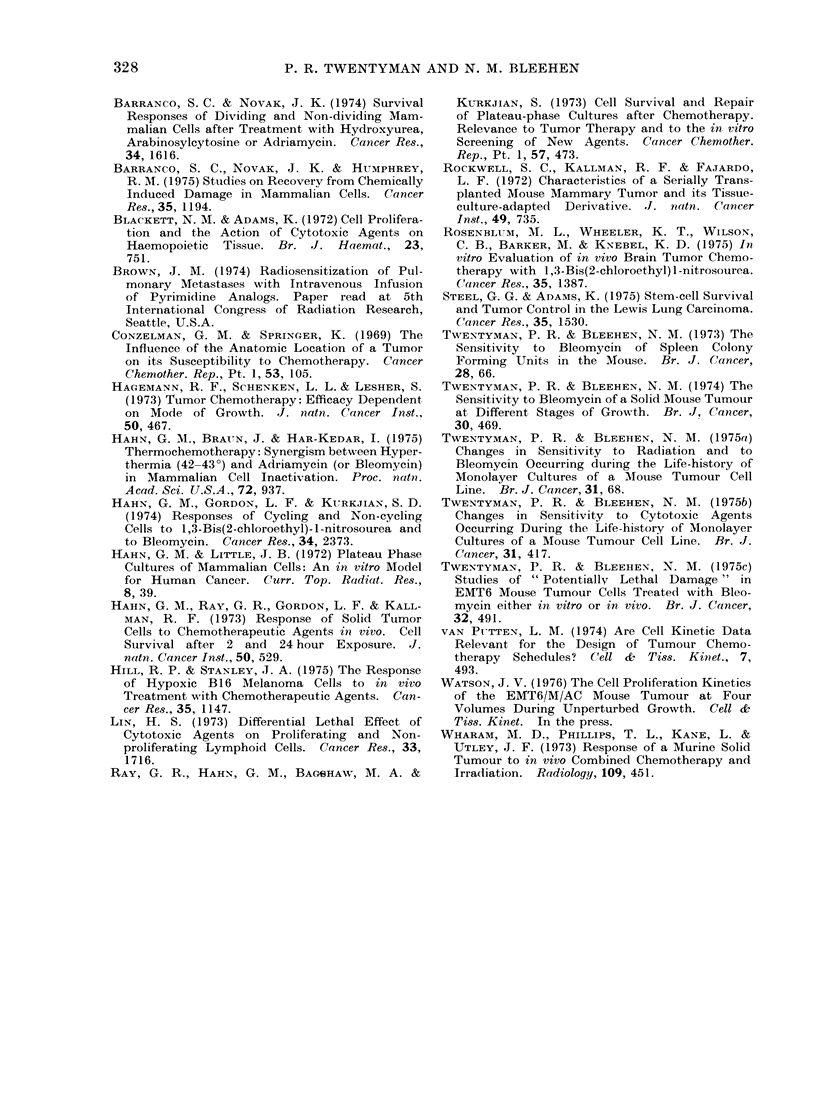# The sensitivity to cytotoxic agents of the EMT6 tumor in vivo. Comparative response of lung nodules in rapid expotential growth and of the solid flank tumour.

**DOI:** 10.1038/bjc.1976.46

**Published:** 1976-03

**Authors:** P. R. Twentyman, N. M. Bleehen

## Abstract

Experiments are described in which dose-response data have been obtained for EMT6 mouse tumour cells growing in vivo and exposed to various cytotoxic agents. A comparison has been made of the response of solid tumours in the flank and of rapidly growing lung nodules. The results are discussed with regard to their cell kinetic implications and compared with our results for the chemosensitivity of EMT6 cells in exponential and plateau phase growth in vitro.


*Br. J. Cancer* (1976) **33**, 320

### THE SENSITIVITY TO CYTOTOXIC AGENTS OF THE EMT6 TUMOUR *IN VIVO*. COMPARATIVE RESPONSE OF LUNG NODULES IN RAPID EXPONENTIAL GROWTH AND OF THE SOLID FLANK TUMOUR

P. R. TWENTYMAN AND N. M. BLEEHEN

*From the M.R.C. Clinical Oncology and Radiotherapeutics Unit, The Medical School, Hills Road, Cambridge, England*

Received 6 October 1975 Accepted 27 November 1975

**Summary.—Experiments are described in which dose-response data have been obtained for EMT6 mouse tumour cells growing *in vivo* and exposed to various cytotoxic agents. A comparison has been made of the response of solid tumours in the flank and of rapidly growing lung nodules. The results are discussed with regard to their cell kinetic implications and compared with our results for the chemosensitivity of EMT6 cells in exponential and plateau phase growth *in vitro*.**

THERE have been several recent studies in which various groups of workers have compared the sensitivity to cytotoxic drugs of mammalian cells during the exponential and plateau phases of growth. (Barranco, Novak and Humphrey, 1973; Barranco and Novak, 1974; Twentyman and Bleehen, 1975*a*, *b*; Hahn, Gordon and Kurkjian, 1974; Ray *et al.*, 1973). The interest in this type of study has been based on the fact that certain similarities exist between the cell proliferation kinetics of plateau phase cultures and the kinetics of experimental solid tumours (Hahn and Little, 1972). Many of the results obtained have been conflicting and this has made it difficult to draw any useful implication for tumour therapy from the data obtained. It has, amongst other things, been difficult to know to what extent the results are determined by cell kinetic differences between exponential and plateau phase cultures and to what extent artefacts of the *in vitro* situation are involved.

We have therefore carried out experiments to compare the sensitivity to cytotoxic drugs *in vivo* of the EMT6 cell line either during rapid exponential growth in the lungs (Brown, 1974) or else growing more slowly as a solid flank tumour. The results of these studies are reported in this paper.

### MATERIALS AND METHODS

The EMT6 cell line may be grown either *in vivo* as a solid tumour or *in vitro* as a monolayer (Rockwell, Kallman and Fajardo, 1972). In addition, assay of cell survival following treatment *in vivo* may be carried out by *in vitro* plating. The particular subline of the tumour used in these experiments was designated EMT6/VJ/AC and was originally supplied to us by Dr E. Frindel. The line is maintained by alternating growth as a solid tumour *in vivo*, and four passages *in vitro*.

For experiments, male Balb/C mice between 10 and 14 weeks of age were inoculated with cells taken from the second, third and fourth *in vitro* passage since removal from a previous *in vivo* passage. For solid flank tumours, $4 \times 10^4$ cells were inoculated intradermally in a volume of 0·05 ml of complete culture medium. For growth as lung nodules, $10^5$ cells were injected into the lateral tail vein in a volume of 0·25 ml of Hanks' solution. The growth of solid tumours was monitored using calipers. Three diameters, mutually at right angles, were measured, and the tumour volume was

TABLE I.—*Cytotoxic Agents Studied*

| Drug name | Source | Method of preparation and administration |
|---|---|---|
| Adriamycin (ADM) | Pharmitalia (UK) Ltd, Barnet, England | Dissolved in sterile water. Injected i.p. in a volume of 0·1 to 0·6 ml |
| Bleomycin (BLM) | Lundbeck Ltd, Luton, England | Dissolved in sterile Hanks' solution. Injected i.p. in a volume of 0·5 ml |
| Cyclophosphamide (Cy) | Ward Blenkinsop Pharmaceuticals Ltd, London, England | Dissolved in sterile water. Injected i.p. in a volume of 0·1 to 0·6 ml |
| 1,3 Bis (2-Chloroethyl)-1-nitrosourea (BCNU) | U.S. National Cancer Inst. | Dissolved to 20 mg/ml in absolute ethanol. Diluted 1 : 20 in sterile Hanks' solution. Injected i.p. in a volume of 0·15 to 0·8 ml |
| 1-(2-Chloroethyl)-3-cyclohexyl-1-nitrosourea (CCNU) | U.S. National Cancer Inst. | Suspended in 0·5% carboxymethyl cellulose. Mixed for 5 min on a laboratory blender. Injected i.p. in a volume of 0·15 to 0·8 ml |

calculated according to the equation derived by Watson (1976).

Preparation of a single cell suspension from solid tumours and *in vitro* assay for surviving fraction was carried out as previously described (Twentyman and Bleehen, 1974, 1975*c*). For lung nodules, the method was virtually identical except that for each complete set of lungs a volume of 15 ml of trypsinized Hanks' solution was used because of the greater mass of tissue involved.

In order to obtain the growth curve for lung nodules, lungs were removed from mice at various times after inoculation of $10^5$ cells and various proportions of each set of lungs were plated out. From the number of colonies produced *in vitro*, it was possible to obtain a value for "dish colonies/set of lungs". From Day 8 onwards, the number of EMT6 cells in the cell suspension was sufficiently large to enable a haemacytometer count to be performed. The tumour cells were very easily distinguishable from the much smaller lung cells.

Two experiments were carried out in which much smaller numbers of EMT6 cells were given intravenously to groups of 6 mice and allowed to develop for 14 days in the lungs. At the end of this time, the lungs were removed, fixed in Carnoy fluid and the lung nodules counted. An experiment was also carried out in which various proportions of normal lungs were plated out with a fixed number of tumour cells in order to see whether the number of colonies produced *in vitro* was dependent upon the number of lung cells present.

Cytotoxic drugs were obtained, prepared and administered as shown in Table I. For dose–response curves, animals were killed at 2 h after drug administration. For time–response curves, times of 30 min, 2 h and 6 h were used. All experiments were carried out at 9 or 10 days following inoculation of tumour cells. Each point in the figures represents a separate determination using pooled cells from two mice with tumours of similar size (for flank tumours) or at the same time after inoculation (for lung nodules).

### RESULTS

### *Growth of flank tumours* (Fig. 1)

A typical growth curve obtained during the recent series of experiments is shown. This is for a group of 8 mice. The tangent to the curve at 9·5 days after inoculation gives a tumour doubling time of around 44 h. This figure is very similar to the value of 46 h which has been estimated from an earlier growth curve obtained for this tumour sub-line and based on 40 animals.

### *Growth of lung nodules* (Fig. 2)

The growth curve for number of dish colonies per set of lungs is shown in Figure 2, and is based on results for individual mice in 4 separate experiments. In individual experiments, the curve was exponential until at least Day 12. Most animals allowed to remain intact died on Days 14–16. It is interesting to note that on Days 8–12, when tumour cells could be counted on a haemacytometer, a relationship between cells plated and dish colonies produced gave a plating

FIG. 1.—Increase in volume of EMT6 flank tumours with time after inoculation of $4 \times 10^4$ cells. Points indicate mean volume for a group of 8 mice and errors shown are the standard error of the mean.

FIG. 2.—Increase in dish colonies per set of lungs plated with time after inoculation of $10^5$ EMT6 cells into the tail vein. Data shown are from 4 separate experiments. Points indicate the mean value for the number of mice shown in parentheses. Error bars show the standard error of the mean.

efficiency of 30–50%. This is similar to our typical value for cells obtained from solid tumours. If it is assumed that the cell yield of our trypsinization method is the same for nodules of all sizes (and we have no reason to believe otherwise), then the line drawn through the points in Figure 2 indicates a doubling time of 17·6 h for the tumour cell population in the lungs. This value is similar to the measured cell cycle time within the proliferating compartment of EMT6 solid tumours. Rockwell *et al.* (1972) obtained a value of 20·7 h for tumours of 200 $mm^3$ volume, and Watson (1976) studying tumours ranging from 1·5 to 175 $mm^3$ obtained a range of cycle times from 14 to 18·5 h. It therefore appears likely that our figure of 17·6 h represents the approximate cycle time of an exponentially growing cell population with a growth fraction of near to 100%. We are currently carrying out a cell kinetic analysis of nodule growth using tritiated thymidine autoradiography.

*Lung nodules/cell inoculated*

In the first experiment where $10^4$ cells were inoculated, the number of lung nodules produced was $37{\cdot}7 \pm 2{\cdot}5$ (one standard error). In the second experiment, where $2 \times 10^3$ cells were inoculated, the number of nodules was $4{\cdot}8 \pm 0{\cdot}6$. These results give figures of 377 and 240 nodules per $10^5$ cells inoculated respectively. The mean value is therefore around 300 nodules per $10^5$ cells. From Figure 2 it may be seen that at Days 9–10 after inoculation of $10^5$ cells, around $10^6$ dish colonies were obtained per set of lungs. If a value of 30–35% is assumed for the plating efficiency, then the number of tumour cells is around $3 \times 10^6$ per set of lungs. By dividing this figure by 300 (the number of nodules per $10^5$ cells inoculated), we obtain a value of about $10^3$ cells per nodule at the time at which experiments were performed. This implies a nodule diameter of 120–150 $\mu$m at this time.

*Effect of lung cells on plating of tumour cells*

It was found that the number of colonies produced *in vitro* by 300 tumour cells plated did not vary with the addition of between 0·01% and 10% of one set of lungs per dish. At 10%, however, there was a light overgrowth of fibroblasts which made the counting of colonies more difficult. A later test showed that at 20% of a set of lungs/dish the dense fibroblast layer made identification of colonies difficult. In experiments, therefore, 10% of one set of lungs/dish has been regarded as the upper acceptable limit in determining the survival of tumour cells at low surviving fractions.

*Response to cytotoxic drugs*

*Bleomycin* (*BLM*).—We have previously shown (Twentyman and Bleehen, 1974) that for the EMT6 solid tumour over a wide range of sizes, the 2 h dose–response curve has virtually reached a plateau of survival at 1 mg/kg and that this plateau continues until at least 10 mg/kg. Furthermore, we have also shown that tumours of all sizes appear to "repair potentially lethal damage" if assay is delayed until 24 h following drug administration. More recently, we have shown that the measured surviving fraction is at a minimum about 30 min after BLM administration and that "repair" is virtually complete by 6 h (Twentyman and Bleehen, 1975*c*). In the current series of experiments we have, therefore, chosen a dose of BLM (4 mg/kg) which is well on to the plateau of the dose–response curve and examined the time response of the solid tumour and

FIG. 3.—Change in surviving fraction of EMT6 cells with time after administration of BLM (4 mg/kg). Closed symbols—flank tumours. Open symbols—lung nodules. The line is drawn to fit the closed symbols only. Errors within individual experiments are small compared with the spread of results between different experiments (except at the 6 h point where a typical 2 × standard error bar is shown).

of lung nodules to this dose. The results are shown in Fig. 3. The responses of the two systems show very similar patterns. Mean surviving fractions at 30 min are about $4 \times 10^{-3}$ for solid tumours and $1 \cdot 3 \times 10^{-2}$ for lung nodules. By 6 h the values have recovered to around 90% and 30% respectively.

*Adriamycin* (*ADM*).—For each of the remaining agents we were anxious to ascertain that the "repair of potentially lethal damage" phenomenon did not operate in such a way as to dictate the time that should be used for a dose–response curve. Preliminary time–response curves for the solid tumour were therefore obtained for each agent. For ADM (60 mg/kg) little decrease in surviving

FIG. 4.—Change in surviving fraction of EMT6 cells with dose of ADM administered 2 h previously. Closed symbols—flank tumours. Open symbols—lung nodules. The line is drawn to fit the closed symbols only. Errors within individual experiments are small compared with the spread of results between different experiments.

FIG. 5.—Change in surviving fraction of EMT6 cells with dose of Cy administered 2 h previously. Closed symbols—flank tumours. Open symbols—lung nodules. The line is drawn to fit the closed symbols only. Errors within individual experiments are small compared with the spread of results between different experiments.

fraction is seen at any time. The 2 h dose-response curves for solid tumour and lung nodules are shown in Fig. 4. Again, there is little effect on the solid tumour at any dose. The effect on lung nodules appears to be slightly greater although, even at the highest doses used, the mean surviving fraction is still around 30%.

*Cyclophosphamide* (*Cy*).—A time-response curve for this agent was carried out at a dose of 60 mg/kg. The mean surviving fraction was a little lower at 2 h than at 30 min or 6 h but not significantly so, given the spread of values obtained. The 2 h dose–response curves are shown in Fig. 5. There is apparently no significant difference between the response of lung nodules and the solid tumour.

*BCNU*.—Following a single dose of BCNU (6 mg/kg) the surviving fractions measured at 2 h and 6 h are similar, and generally lower than the 30 min values. The 2 h dose–response curves are shown in Fig. 6. The responses are similar although there is perhaps a trend towards a slightly greater response for lung nodules at higher doses.

*CCNU*.—The time-response curve for this agent was carried out at a dose of 20 mg/kg. The mean value of surviving fraction observed at 6 h was similar to the 2 h value, and much lower than the surviving fraction at 30 min. The 2 h dose–response curves are shown in Fig. 7. There is a very wide spread of results for this agent. This is probably due to the method of drug administration with consequent variability in drug

FIG. 6.—Change in surviving fraction of EMT6 cells with dose of BCNU administered 2 h previously. Closed symbols—flank tumours. Open symbols—lung nodules. The line is drawn to fit the closed symbols only. Errors within individual experiments are small compared with the spread of results between different experiments.

FIG. 7.—Change in surviving fraction of EMT6 cells with dose of CCNU administered 2 h previously. Closed symbols—flank tumours. Open symbols—lung nodules. The line is drawn to fit the closed symbols only. Errors within individual experiments are small compared with the spread of results between different experiments.

absorption. Again it does not appear that there is any significant difference in the response of the two systems.

### DISCUSSION

Before consideration of any cell kinetic implications of our results it is necessary to point out that the cells under study in our two systems are not exposed to uniform concentrations of drugs. It is difficult to make realistic estimates of how drug concentrations will vary but it is generally accepted that there are populations of cells in solid tumours which are relatively distant from blood vessels and hence less accessible to substances dissolved in the blood stream. Should uneven drug availability be a factor in these studies, therefore, it would probably be expected to be shown by the presence of a resistant fraction of cells in the solid flank tumours.

*Bleomycin.*—Interpretation of BLM response curves where cell survival is assayed by transplantation techniques is extremely difficult because of the operation of the effect usually referred to as "repair of potentially lethal damage". We have discussed this effect fully in a recent publication (Twentyman and Bleehen, 1975*c*). What is very obvious from the results, however, is that this effect operates to nearly the same extent in lung nodules as it does in the solid tumour. If, therefore, this effect of delayed subculture *in vivo* operates via the same mechanism as that *in vitro*, our finding would agree with the observation that the ability to repair potentially lethal damage is possessed by both exponential and plateau phase cells *in vitro* (Barranco *et al.*, 1975; Twentyman and Bleehen, 1975*c*) rather than being confined to plateau phase cells (Ray *et al.*, 1973). The results reported here are consistent with our observation (Twentyman and Bleehen, 1973) that proliferating spleen colony-forming cells in mouse marrow are more sensitive than are quiescent CFUs to BLM when assay is carried out at 24 h after drug administration, although it is possible that a different conclusion may have been reached had the assay been performed at a different time.

*Adriamycin.*—Our previously reported results for the response of exponential and plateau phase cells *in vitro* to ADM (Twentyman and Bleehen, 1975*b*) are similar to those reported by Barranco and Novak (1974). The response for exponential phase cells is very rapid, falling to $10^{-3}$ for a dose of 1 $\mu$g/ml for 1 h, whereas the response of plateau phase cells is very much less, the surviving fraction being in excess of $10^{-1}$ at 1 $\mu$g/ml. If these *in vitro* results could be directly applied to the *in vivo* situation, then one would expect the dose-response curves for lung nodules and solid tumours to be very different. In fact, both systems are extremely insensitive to even very high doses of this agent. This could, of course, be due to the drug not reaching the cells when given by the i.p. route. However, Hahn, Braun and Har-Kedar (1975) have found the response of the EMT6 tumour to adriamycin to be only a little increased if the drug is given by the intravenous instead of the intraperitoneal route. Furthermore, these authors found that the response can be extremely severe if the tumour is heated, due, at least in part, to increased penetration of drug into the cell. It would therefore appear that access of ADM into the cell is the problem here rather than availability of drug in the extracellular environment. If this is so, then the very rapid response shown by exponential cells *in vitro* appears not to be reflected *in vivo* and may be only indicative of the state of the cell membrane during this particular phase of *in vitro* growth.

*Cyclophosphamide.*—Our results suggest that there is no great change in the measured surviving fraction between 2 and 6 h following administration of Cy. It appears therefore that for the purposes of our present study, a time of 2 h after drug administration is appropriate for comparison of dose–response data. Repair of potentially lethal drug damage may, however, occur between 6 and 24 h (Hahn *et al.*, 1973).

The dose–response curves for cyclophosphamide do not indicate any difference in sensitivity between cells in lung nodules and in solid tumours. This suggests agreement with the results of Wharam *et al.* (1973), who found that Cy has an equal effect against oxygenated and hypoxic cells in the EMT6 tumour and also the recent results of Hill and Stanley (1975) who reported a similar finding for the B16 melanoma. Also, Blackett and Adams (1972) found little difference in the sensitivity of slowly and rapidly proliferating cells in the repopulating compartment of the erythroid series. On the other hand Steel and Adams (1975) have recently shown that the response to Cy of small lung nodules of the Lewis lung carcinoma may be greater than that of larger tumours, Lin (1973) showed a marked difference in the response of slowly and rapidly proliferating lymphoid cells to Cy, and van Putten (1974) has shown a degree of proliferation dependence for spleen colony-forming units in the mouse marrow. It would therefore appear that the conclusion regarding the proliferation dependence of cyclophosphamide depends greatly upon the cell type studied. In situations where repair of potentially lethal damage may operate, and probably to different extents in the cycling and non-cycling populations, the conclusions regarding differential sensitivity may well be dependent upon the timing of the experiment. It seems, however, that the anoxic (and probably non-cycling) compartment of cells in solid tumours probably does not represent a population resistant to this drug.

*BCNU.*—The data of Barranco *et al.* (1975) indicate that *in vitro* there is no recovery from potentially lethal damage by cells treated with this agent. Our *in vivo* data (Fig. 7) and those of Hahn *et al.* (1974) support this idea. The dose–response curve we have obtained for the solid tumour appears to be almost exponential down to a surviving fraction of $10^{-4}$ at a dose of 25–30 mg/kg. This is similar in shape but a little more steep than the curve obtained for the B16 melanoma by Hill and Stanley (1975). On the other hand, in a recent study of the response to BCNU of cells in a transplanted brain tumour in the rat, Rosenblum *et al.* (1975) found a very resistant fraction of about $10^{-3}$ of the total population. Also, in a study of the P815X2 mastocytoma in the mouse, the tumour cells were found by Hagemann, Schenken and Lesher (1973) to be very resistant to BCNU, and the authors suggest that drug availability is the likely cause. Our data for lung nodules suggest a response curve which is perhaps a little steeper than that for the solid tumour. It would appear therefore, that in our solid tumour system, there is no large population of cells which is resistant to BCNU for any reason. Furthermore, the sensitivity of the cycling and non-cycling compartment appears to be similar. This is in agreement with our findings that EMT6 cells growing *in vitro* show similar sensitivity to BCNU in the exponential, early plateau and late plateau phases of growth (Twentyman and Bleehen, 1975*b*). The data for other tumours, however, indicate that our findings may not apply to all such systems.

*CCNU.*—Absence of repair of potentially lethal damage observed for CCNU in our system is in agreement with the findings *in vitro* of Barranco *et al.* (1975). Again, the sensitivity of EMT6 cells in solid tumours and in lung nodules appears to be similar with no evidence for the presence of a resistant fraction in the solid tumour. This result is in agreement with that of Hill and Stanley (1975) for the B16 melanoma, and also with our *in vitro* results for EMT6 cells in exponential and plateau phase (Twentyman and Bleehen, 1975*b*).

In general, therefore, in our system there does not seem to be much difference in the response of lung nodules and solid tumours to any of the agents studied. This is in contrast to the results of Conzelman and Springer (1969) who found that the SAH1-1 mouse tumour was more sensitive to several drugs when growing subcutaneously than when growing in the lungs.

The results presented in the present paper, notably those for ADM, further emphasize a point we have made in previous publications, i.e. it is dangerous to extrapolate results obtained in one experimental situation to another situation. This applies both to extrapolation from one cell line to another *in vitro* and to extrapolation from the *in vitro* to the *in vivo* situation for the same cell line. Before such comparisons can legitimately be made, we need to know much more about the factors both kinetic and otherwise which determine the response of cells to cytotoxic drugs.

We thank Stella Keller and Karen Day for their technical assistance. Bleomycin and CCNU were kindly supplied by Lundbeck Limited, and BCNU was a gift from the Drug Development Branch, Division of Cancer Treatment of the United States National Cancer Institute.

### REFERENCES

Barranco, S. C., Novak, J. K. & Humphrey, R. M. (1973) Response of Mammalian Cells Following Treatment with Bleomycin and 1,3-Bis(2-chloroethyl)-1-nitrosourea during Plateau Phase. *Cancer Res.*, **33**, 691.

BARRANCO, S. C. & NOVAK, J. K. (1974) Survival Responses of Dividing and Non-dividing Mammalian Cells after Treatment with Hydroxyurea, Arabinosylcytosine or Adriamycin. *Cancer Res.*, **34**, 1616.

BARRANCO, S. C., NOVAK, J. K. & HUMPHREY, R. M. (1975) Studies on Recovery from Chemically Induced Damage in Mammalian Cells. *Cancer Res.*, **35**, 1194.

BLACKETT, N. M. & ADAMS, K. (1972) Cell Proliferation and the Action of Cytotoxic Agents on Haemopoietic Tissue. *Br. J. Haemat.*, **23**, 751.

BROWN, J. M. (1974) Radiosensitization of Pulmonary Metastases with Intravenous Infusion of Pyrimidine Analogs. Paper read at 5th International Congress of Radiation Research, Seattle, U.S.A.

CONZELMAN, G. M. & SPRINGER, K. (1969) The Influence of the Anatomic Location of a Tumor on its Susceptibility to Chemotherapy. *Cancer Chemother. Rep.*, Pt. 1, **53**, 105.

HAGEMANN, R. F., SCHENKEN, L. L. & LESHER, S. (1973) Tumor Chemotherapy: Efficacy Dependent on Mode of Growth. *J. natn. Cancer Inst.*, **50**, 467.

HAHN, G. M., BRAUN, J. & HAR-KEDAR, I. (1975) Thermochemotherapy: Synergism between Hyperthermia (42–43°) and Adriamycin (or Bleomycin) in Mammalian Cell Inactivation. *Proc. natn. Acad. Sci. U.S.A.*, **72**, 937.

HAHN, G. M., GORDON, L. F. & KURKJIAN, S. D. (1974) Responses of Cycling and Non-cycling Cells to 1,3-Bis(2-chloroethyl)-1-nitrosourea and to Bleomycin. *Cancer Res.*, **34**, 2373.

HAHN, G. M. & LITTLE, J. B. (1972) Plateau Phase Cultures of Mammalian Cells: An *in vitro* Model for Human Cancer. *Curr. Top. Radiat. Res.*, **8**, 39.

HAHN, G. M., RAY, G. R., GORDON, L. F. & KALLMAN, R. F. (1973) Response of Solid Tumor Cells to Chemotherapeutic Agents *in vivo*. Cell Survival after 2 and 24 hour Exposure. *J. natn. Cancer Inst.*, **50**, 529.

HILL, R. P. & STANLEY, J. A. (1975) The Response of Hypoxic B16 Melanoma Cells to *in vivo* Treatment with Chemotherapeutic Agents. *Cancer Res.*, **35**, 1147.

LIN, H. S. (1973) Differential Lethal Effect of Cytotoxic Agents on Proliferating and Non-proliferating Lymphoid Cells. *Cancer Res.*, **33**, 1716.

RAY, G. R., HAHN, G. M., BAGSHAW, M. A. & KURKJIAN, S. (1973) Cell Survival and Repair of Plateau-phase Cultures after Chemotherapy. Relevance to Tumor Therapy and to the *in vitro* Screening of New Agents. *Cancer Chemother. Rep.*, Pt. 1, **57**, 473.

ROCKWELL, S. C., KALLMAN, R. F. & FAJARDO, L. F. (1972) Characteristics of a Serially Transplanted Mouse Mammary Tumor and its Tissue-culture-adapted Derivative. *J. natn. Cancer Inst.*, **49**, 735.

ROSENBLUM, M. L., WHEELER, K. T., WILSON, C. B., BARKER, M. & KNEBEL, K. D. (1975) *In vitro* Evaluation of *in vivo* Brain Tumor Chemotherapy with 1,3-Bis(2-chloroethyl)1-nitrosourea. *Cancer Res.*, **35**, 1387.

STEEL, G. G. & ADAMS, K. (1975) Stem-cell Survival and Tumor Control in the Lewis Lung Carcinoma. *Cancer Res.*, **35**, 1530.

TWENTYMAN, P. R. & BLEEHEN, N. M. (1973) The Sensitivity to Bleomycin of Spleen Colony Forming Units in the Mouse. *Br. J. Cancer*, **28**, 66.

TWENTYMAN, P. R. & BLEEHEN, N. M. (1974) The Sensitivity to Bleomycin of a Solid Mouse Tumour at Different Stages of Growth. *Br. J, Cancer*, **30**, 469.

TWENTYMAN, P. R. & BLEEHEN, N. M. (1975*a*) Changes in Sensitivity to Radiation and to Bleomycin Occurring during the Life-history of Monolayer Cultures of a Mouse Tumour Cell Line. *Br. J. Cancer*, **31**, 68.

TWENTYMAN, P. R. & BLEEHEN, N. M. (1975*b*) Changes in Sensitivity to Cytotoxic Agents Occurring During the Life-history of Monolayer Cultures of a Mouse Tumour Cell Line. *Br. J. Cancer*, **31**, 417.

TWENTYMAN, P. R. & BLEEHEN, N. M. (1975*c*) Studies of "Potentially Lethal Damage" in EMT6 Mouse Tumour Cells Treated with Bleomycin either *in vitro* or *in vivo*. *Br. J. Cancer*, **32**, 491.

VAN PUTTEN, L. M. (1974) Are Cell Kinetic Data Relevant for the Design of Tumour Chemotherapy Schedules? *Cell & Tiss. Kinet.*, **7**, 493.

WATSON, J. V. (1976) The Cell Proliferation Kinetics of the EMT6/M/AC Mouse Tumour at Four Volumes During Unperturbed Growth. *Cell & Tiss. Kinet.* In the press.

WHARAM, M. D., PHILLIPS, T. L., KANE, L. & UTLEY, J. F. (1973) Response of a Murine Solid Tumour to *in vivo* Combined Chemotherapy and Irradiation. *Radiology*, **109**, 451.